# GLP-1 Receptor Agonists as a Novel Solution for Antipsychotic-Induced Weight Gain in Severe and Persistent Mental Illness

**DOI:** 10.1177/07067437251386626

**Published:** 2025-10-15

**Authors:** Samantha Jacobson, Noah Margolese, Howard C. Margolese

**Affiliations:** 1 Faculty of Medicine, 12370 University of Sherbrooke, Sherbrooke, Canada; 2 Department of Psychiatry, 54473 McGill University Health Centre, Montreal, Canada

**Keywords:** adherence, antipsychotics, healthcare policy, schizophrenia

## Abstract

Patients with severe and persistent mental illness (SPMI) experience significant metabolic side effects from antipsychotic medications, including antipsychotic-induced weight gain (AIWG). This contributes to a high prevalence of obesity, insulin resistance, and type 2 diabetes in this population, ultimately reducing life expectancy. Traditional weight management strategies, such as behavioural interventions, are often less feasible in this group. Glucagon-like peptide-1 receptor agonists (GLP-1RAs), initially developed for type 2 diabetes, have shown promise in addressing AIWG by reducing weight, improving metabolic parameters, and offering potential neuroprotective and psychiatric benefits. Evidence supports the efficacy of GLP-1RAs in managing AIWG, with studies demonstrating substantial reductions in weight and body mass index without exacerbating psychiatric symptoms. However, access to these medications remains limited due to high costs and restrictive healthcare policies. Expanding access to GLP-1RAs could bridge a critical gap in care for patients with SPMI, improving both physical and mental health outcomes. Future research should focus on evaluating long-term efficacy and cost-effectiveness, particularly in the Canadian healthcare context, to inform policy changes and optimize treatment strategies.

**Plain Language Summary Title:**

Can diabetes medications help treat weight gain caused by antipsychotics?

## Plain Language Summary

People with serious mental illnesses, like schizophrenia, often take antipsychotic medications. While these drugs are important for managing symptoms, many cause rapid weight gain. This side effect can lead to physical health problems, worsen mental health, and make people stop taking their medication. Unfortunately, current weight-loss strategies are often unrealistic for people with severe mental illness. This study explores whether a group of medications called GLP-1 receptor agonists, originally developed to treat diabetes, could help reduce this medication-related weight gain. GLP-1RAs led to meaningful weight loss, even in patients who hadn't responded to other medications like metformin. These drugs also helped lower blood sugar and improve other health markers. Some studies even showed that GLP-1RAs might reduce psychiatric symptoms and risks of conditions like substance use, suicide, and dementia. However, side effects such as nausea and vomiting were common, especially at the beginning of treatment. Access remains limited due to high costs and lack of public insurance coverage in many regions. This highlights a new way to support people who struggle with weight gain from antipsychotics. If made more accessible, GLP-1RAs could help patients stay on their medications, improve both mental and physical health, and reduce long-term healthcare costs. More studies are needed, especially in psychiatric populations, but this treatment option shows promise for improving quality of life in a group that often faces major health inequalities.

## What are GLP-1 Receptor Agonists?

Glucagon-like peptide-1 receptor agonists (GLP-1RAs) are medications initially designed to treat type 2 diabetes mellitus (T2DM) by regulating blood glucose levels.^1^ These drugs work by mimicking the effects of the glucagon-like peptide-1 (GLP-1) hormone, thereby slowing gastric emptying, increasing satiety, and reducing appetite.^1^ The primary advantages of GLP-1RAs lie in glucose reduction, cardiorenal benefits, and amelioration of metabolic syndrome.^1^ Furthermore, GLP-1RAs cause weight reduction by facilitating glucose uptake and suppressing appetite. These medications are now used to counteract metabolic disturbances, such as weight gain and insulin resistance, which are frequently associated with antipsychotics.^1^

## Why is it Important to Address AIWG in Patients With SPMI?

Patients with severe and persistent mental illness (SPMI) have a 10–15-year shorter life expectancy than the general population.^2^ This shortened lifespan is due to various factors, including dysmetabolic symptoms, including obesity, and insulin resistance, which are significant risk factors linked to increased cardiovascular risk and premature mortality.^2^ Other factors include low socioeconomic status, unhealthy lifestyles, and inadequate healthcare.^3^ Many antipsychotics are associated with clinically significant weight gain and metabolic disturbances, including the development of T2DM.^4^ This is particularly true for olanzapine and clozapine, which have been linked to rapid and severe weight gain.^4^ Patients with SPMI often experience the dual stigma of living with both mental illness and obesity. This stigma can affect their self-esteem, and willingness to seek help, making the management of antipsychotic-induced weight gain (AIWG) even more challenging. Healthcare providers may unconsciously reinforce biases about obesity in SPMI patients by assuming they are noncompliant, unmotivated, or responsible for their weight.^3^ These implicit biases can adversely affect clinical decision-making and treatment planning,^3^ and may manifest as diminished trust, poorer therapeutic alliances, and inequitable care.

AIWG also contributes to worse mental health outcomes and antipsychotic medication discontinuation. A qualitative descriptive study of individuals with AIWG found that many self-reduced or stopped antipsychotic treatment in an effort to reverse AIWG.^5^ This can result in relapses, further exacerbating their illnesses. Existing guidelines on weight management often emphasize behavioural interventions that may be difficult for patients with SPMI, particularly given the motivational challenges they face.^5^ In contrast, participants viewed pharmacological adjuncts as relevant and acceptable and requested that these be offered earlier than currently endorsed.^5^ Evidence-based pharmacological interventions, including GLP-1RAs, can complement lifestyle interventions and provide a comprehensive solution. Current guidelines recommend metformin as the first-line agent for AIWG. While metformin has shown modest efficacy in reducing weight and improving metabolic parameters, particularly in first-episode or younger patients, some may not achieve sufficient response or tolerate metformin.^6^ In this context, GLP-1RAs represent a promising adjunctive or second-line option.

## How Effective are GLP-1 Receptor Agonists?

GLP-1RAs have shown substantial benefits in managing obesity in multiple large randomized controlled trials, as summarized in Table 1. In the STEP 1 trial, once-weekly semaglutide 2.4 mg combined with lifestyle intervention led to mean weight loss of 14.9% over 68 weeks, compared to 2.4% with placebo, with 86.4% achieving at least 5% weight loss.^7^ STEP 3, which included intensive behavioural therapy and a low-calorie diet, demonstrated weight loss of 16.0% versus 5.7% with placebo, alongside improvements in waist circumference and physical functioning.^8^ In STEP 4, participants who continued semaglutide 2.4 mg maintained significantly greater weight loss (−7.9%) compared to those switched to placebo with additional benefits including reductions in waist circumference (−9.7 cm), systolic blood pressure (−3.9 mmHg), and improvements in physical functioning scores.^9^ Long-term efficacy was confirmed in STEP 5, where 15.2% weight loss was maintained over 104 weeks.^10^ In STEP 8 semaglutide caused a mean body weight reduction of 15.8%, representing an average loss of over 15 kg.^11^ Most participants (70.9%) achieved ≥10% weight loss, and over half (55.6%)  ≥ 15% weight loss, thresholds associated with significant metabolic and quality-of-life improvements.^11^ Similarly, SUSTAIN (notably SUSTAIN-3^12^) evaluated semaglutide in patients with T2DM and showed reductions in HbA1c (−1.5%) and weight (−5.6 kg) compared to exenatide XR (−0.9%, −1.9 kg).^12^ SURMOUNT-1 evaluated 15 mg once-weekly tirzepatide in 2,539 adults with obesity and no diabetes, and reported mean weight loss of 20.9% (−22.5 kg) over 72 weeks. Even 5 mg tirzepatide led to 15.0% body weight reduction.^13^ Taken together, data from these trials establish semaglutide and tirzepatide as highly effective in individuals with obesity or T2DM. However, they excluded individuals with SPMI. Data regarding semaglutide in individuals with SPMI remain limited.

**Table 1.** Key Randomized Controlled Trials Evaluating the Efficacy and Safety of Semaglutide in Obesity Treatment.

Study	Design	Intervention	Comparator	N (I/C)	Population	Duration	Weight loss	Other benefits	Adverse effects
SUSTAIN-3 (Aroda et al., 2019)^12^	DB-RCT	Semaglutide 1 mg weekly	Exenatide XR 2 mg weekly	813 / 410	T2DM patients on oral antidiabetics	56 weeks	−5.6 kg vs −1.9 kg (Δ −3.7 kg; *P* < 0.0001)	HbA1c −1.5%, ↓BP and lipids	GI AEs: 41.8% vs. 33.3%; injection-site reactions: 1.2% vs. 22%
STEP 1 (Wilding et al., 2021)^7^	DB-RCT	Semaglutide 2.4 mg weekly	Placebo	1306 / 655	Adults with BMI ≥30 or ≥27 with ≥1 comorbidity, no diabetes	68 weeks	-14.9% vs -2.4%	86% ≥ 5% WL, 69% ≥ 10% WL 50.5% ≥ 15%; ↓cardiometabolic risk factors, ↑physical functioning	GI AEs 74.2% vs. 47.9%; D/C 4.5% vs. 0.8%
STEP 3 (Wadden et al., 2021)^8^	DB-RCT	Semaglutide 2.4 mg weekly + therapy	Placebo + behavioural therapy	611 / 204	Adults with BMI ≥30 or ≥27 with ≥1 comorbidity, no diabetes	68 weeks	-16.0% vs -5.7% (Δ 10.3%, *P* < 0.001)	89% ≥ 5% WL, 75% ≥ 10% WL	GI AEs in 82.8% semaglutide vs. 63.2% placebo; D/C 3.4%
STEP 4 (Rubino et al., 2021)^9^	DB-RCT	Continued semaglutide 2.4 mg once weekly + lifestyle intervention	Switch to placebo + lifestyle intervention	535 / 268	Adults with BMI ≥30 or ≥27 with ≥1 comorbidity, no diabetes	68 weeks	-7.9% vs +6.9% (Δ: -14.8%, *P* < 0.001)	↓Waist circumference (-9.7 cm), ↓SBP (-3.9 mmHg), ↑Physical functioning score (SF-36: + 2.5)	GI AEs in 49.1% vs. 26.1%; D/C 2.4% vs. 2.2%
STEP 5 (Garvey et al., 2022)^10^	DB-RCT	Semaglutide 2.4 mg weekly	Placebo	152/152	Adults with obesity or BMI ≥27 + ≥1 comorbidity, no diabetes	104 weeks	−15.2% vs. −2.6% (Δ −12.6%, *P* < 0.0001)	77.1% ≥ 5% WL vs. 34.4%	GI AEs in 82.2% vs. 53.9%, mostly mild-to-moderate
STEP 8 (Rubino et al., 2022)^11^	DB-RCT,	Semaglutide 2.4 mg weekly + diet/activity	Liraglutide 3.0 mg daily + diet/activity	126 / 127	Adults with obesity or overweight, no diabetes	68 weeks	−15.8% vs −6.4% (Δ −9.4%, *P* < 0.001)	≥10% WL in 70.9%, ≥15% in 55.6%	GI AEs: 84.1% (semaglutide), 82.7% (liraglutide); D/C: 13.5% vs 27.6%
SURMOUNT-1 (Jastreboff et al., 2022)^13^	DB-RCT, 119 sites	Tirzepatide 5–15 mg weekly	Placebo	1905/ 634	Adults with BMI ≥30 or ≥27 + ≥1 comorbidity, no diabetes	72 weeks	-15.0% (5 mg), -19.5% (10 mg), -20.9% (15 mg) vs. -3.1% (*P* < 0.001	≥20% WL in 50% (15 mg)	GI AEs common (mild/moderate), D/C due to AEs: 4.3% (5 mg), 7.1% (10 mg), 6.2% (15 mg), 2.6% (placebo)

DB-RCT: double blind-randomized control trial; WL: weight loss; D/C: discontinued; SBP: systolic blood pressure; AEs: adverse events.

COaST^14^ is the first randomized controlled trial of semaglutide in individuals with schizophrenia treated with clozapine. In this 36-week, double-blind study, 31 were randomized to receive once-weekly semaglutide 2.0 mg or placebo. Semaglutide led to mean weight loss of 13.88%, compared to 0.42% with placebo (*P* < 0.0001). Clinically significant weight loss was achieved by 93.3% (≥5% loss) and 66.7% (≥10% loss). Importantly, semaglutide did not exacerbate psychotic symptoms, nor alter clozapine or norclozapine plasma levels supporting its safety in this high-risk group. The tolerability profile was consistent with prior studies, with mild gastrointestinal effects being the most common side effect. These results suggest that semaglutide may offer superior efficacy compared to metformin in patients with clozapine-induced weight gain and underscores its potential in this population.

A Canadian case series evaluated semaglutide in patients with AIWG who had not responded to metformin.^15^ Over 12 months, 12 patients experienced a mean weight loss of 8.67 ± 9.0 kg (*P* = 0.04), with 41.6% losing over 7% body weight.^15^ Gastrointestinal side-effects were mild and transient, and no serious adverse events were reported. Bak et al.^16^ conducted a systematic review of GLP-1RAs, specifically exenatide and liraglutide, in managing AIWG in schizophrenia or schizoaffective disorder. In 12 to 24 weeks, liraglutide led to significant weight loss of 4.70 kg (95%CI: −4.85 to −4.56; *P* < 0.001), while exenatide reduced mean weight by 2.48 kg (95%CI: −5.12 to +0.64; *P* = 0.07).^16^ Both medications significantly reduced body mass index (BMI), liraglutide by 1.52 kg/m² (95%CI: −1.83 to −1.22; *P* < 0.001) and exenatide 0.82 kg/m² (95%CI: −1.56 to −0.09; *P* = 0.03).^16^ In a systematic review that included 36-studies GLP-1RAs (especially liraglutide, semaglutide, and exenatide) were consistently associated with reduced body weight, BMI, waist circumference, fasting glucose, and HbA1c in patients taking second-generation antipsychotics.^17^ Importantly, GLP-1RAs were not associated with worsened psychiatric symptoms, and in some studies, were linked to improved depressive symptoms, quality-of-life, and global functioning scores.^17^ Notably, semaglutide may potentially reduce alcohol consumption and reward-related brain activation in patients with alcohol use disorder.^17^ However, the most compelling arguments for GLP-1RAs extends beyond weight management. By reducing AIWG, a key factor in treatment discontinuation, as shown in a recent systematic review and meta-analysis,^18^ GLP-1RAs may improve antipsychotic adherence in patients with SPMI.

Xie et al.^19^ evaluated the effectiveness and risks of GLP-1RAs compared to other antihyperglycemics in 1,960,067 individuals using US Veterans Affairs databases. GLP-1RA use was linked to lower risks of substance-related disorders, including alcohol (hazard ratio [HR] 0.89, 95% confidence interval [CI] 0.86−0.92), cannabis (0.88 [0.83−0.93]), stimulant (0.84 [0.78−0.91]), and opioid use disorders (0.87 [0.82−0.92]).^19^ Moreover, GLP-1RAs were associated with reduced suicidal ideation, self-harm (0.90 [0.86−0.94]), bulimia (0.81 [0.77−0.84]), and other psychotic disorders (0.82 [0.76−0.89]).^19^ GLP-1RAs also demonstrated a neuroprotective effect, reducing risk of seizures (0.90 [0.85−0.95]) and neurocognitive disorders (0.95 [0.93−0.97]), including dementia (0.92 [0.88−0.97]) and Alzheimer's disease (0.88 [0.78−0.99]). The breadth of these findings suggests that GLP-1RAs could transform the therapeutic landscape for patients with SPMI by addressing both metabolic and psychiatric comorbidities in a single intervention. However, it is important to note that causality has not been established, and direct effects on psychotic symptoms have not been demonstrated in randomized trials.

Preclinical studies further explore these possibilities. In a murine model of psychosis, liraglutide demonstrated antipsychotic-like effects, becoming the first antidiabetic drug to show such activity in mice.^20^ This effect was not observed with Dipeptidyl peptidase-4 (DPP-4) inhibitors like sitagliptin, which fail to cross the blood-brain barrier (BBB).^20^ Recent work highlights that BBB penetration varies across GLP-1RAs and more studies are needed to elucidate their CNS activity. While the potential for GLP-1RAs to modulate dopamine-mediated processes is intriguing, current evidence remains preliminary and should be interpreted with caution.

These early findings raise the possibility that GLP-1RAs could be repurposed as adjunctive treatments for neuropsychiatric disorders. Their ability to target both metabolic and psychiatric outcomes positions them as promising, though investigational, in the management of complex psychiatric conditions while improving overall health outcomes.

## What are the Harms of Giving GLP-1 Receptor Agonists?

Despite their benefits, GLP-1RAs are associated with potential adverse effects, primarily gastrointestinal symptoms. Bak et al.^16^ reported that nausea was experienced by 38.1% on liraglutide and 40.8% on exenatide. Other common side-effects include vomiting (25.3% liraglutide, 19.8% exenatide) and diarrhoea (13.3% liraglutide, 31.5% exenatide). While liraglutide showed favourable metabolic effects, including improved fasting glucose and reduced LDL cholesterol, exenatide increased LDL by 0.70 mmol/L.^16^ In STEP 8, nausea (39.7% semaglutide vs. 47.4% liraglutide), vomiting (15.2% vs. 16.1%), and diarrhoea (20.3% vs. 22.6%) were commonly reported.^11^ However, semaglutide had fewer treatment discontinuations due to adverse events (13.5%) compared to liraglutide (27.6%), suggesting better tolerability despite similar side-effect profiles.^11^ In SUSTAIN, semaglutide produced similar rates of gastrointestinal adverse events, most commonly nausea and diarrhoea, though most symptoms were transient and mild-to-moderate in severity.^12^ In SURMOUNT-1, gastrointestinal side-effects were again the most frequent.^13^ Nausea (31–36%), diarrhoea (18–21%), and vomiting (17–23%) depended on tirzepatide dose. Most events occurred during dose-escalation and declined afterwards.^13^ Xie et al.^19^ found that tirtazepide increased the risk of hypotension, syncope, arthritic disorders, nephrolithiasis, interstitial nephritis and drug-induced pancreatitis.

Notably, discontinuing GLP-1RAs often leads to weight regain due to hormonal imbalances, β-cell dysfunction, and disrupted central appetite regulation,^1^ necessitating indefinite use to maintain benefits. This raises concerns for SPMI patients, who already face high medication burdens and underscores the need for additional research to assess long-term safety and effectiveness of GLP-1RAs in managing AIWG.

Although GLP-1RAs are not typically associated with drug–drug interactions, their mechanism, delaying gastric emptying, has raised concerns about altered absorption of oral medications.^21^ While these effects have been modelled or observed for several cardiovascular and anticoagulant medications including rosuvastatin, valsartan, and dabigatran,^21^ we were unable to identify any studies evaluating GLP-1RAs impact on pharmacokinetics or clinical efficacy of oral antipsychotics. This remains an important underexplored area for future research.

## What are the Resource Implications?

Currently, accessibility of GLP-1RAs in the psychiatric population is limited. At least one GLP-1RA, semaglutide, is covered by most provincial regimes, however coverage is limited to patients treated for diabetes mellitus. Liraglutide is only covered in Quebec, and exenatide is not covered by any province. Private insurances only sporadically cover GLP-1RAs for other indications. Given that up to 90% of individuals with schizophrenia are unemployed,^22^ private insurance is not a viable solution to bridge this healthcare gap on a population level.

GLP-1RAs are associated with significant costs. At typical maintenance doses liraglutide has an estimated yearly cost of $5,839 per Canadian, and an estimated societal cost of $46,476 per increased Quality-Adjusted Life Year (QALY) when compared to diet and exercise.^23^ While liraglutide was less cost-effective than semaglutide and orlistat, it fell within the $50,000/QALY cutoff.^23^ The comparison to diet and exercise may be less applicable in the context of AIWG due to reduced feasibility in SPMI. These costs, however, must be weighed against those associated with AIWG and antipsychotic nonadherence. Although data on AIWG specifically is lacking, estimates of the economic burden of obesity in Canada range from $4.6 billion to $7.1 billion annually,^24^ while those associated with schizophrenia totalled $6.85 billion in 2004.^25^ By 2017–2018, average cost of hospitalization for a Canadian patient with schizophrenia reached $12,971, making it the most expensive among all mental health conditions.^26^

Beyond Canada, recent international evidence highlights semaglutide as cost-effective and adherence-promoting. An American cost-effectiveness analysis comparing four GLP-1RAs (semaglutide, liraglutide, dulaglutide, and exenatide) found that semaglutide yielded the greatest QALY gain and was most cost-effective.^27^ This result was largely driven by semaglutide's superior weight-loss efficacy and modelled real-world utility. Similarly, a British analysis using the IQVIA Core Diabetes Model determined that once-weekly semaglutide 1 mg was more effective and less costly than once-daily liraglutide 1.8 mg, despite liraglutide's 33% lower price.^28^ These findings support the clinical and economic viability of semaglutide in resource-limited psychiatric settings. Further studies are required to directly evaluate the cost-effectiveness of GLP-1RAs for AIWG in SPMI, including in a Canadian context. Future studies may indicate potential as a cost-effective and clinically effective intervention, especially if reduced relapse and hospitalization rates can be demonstrated.

## What can be Expected in the Foreseeable Future?

As evidence for the efficacy of GLP-1RAs in managing AIWG grows, these treatments may play an increasingly central role not only in addressing the metabolic side-effects of antipsychotics but also in improving psychiatric outcomes for patients with SPMI. Xie et al.^19^ provided compelling evidence that GLP-1RAs are associated with a reduced risk of multiple psychiatric disorders, including substance-related disorders, suicidal ideation, and dementia. This large-scale cohort study underscores the therapeutic potential of GLP-1RAs beyond weight management. Despite these promising findings, access remains limited due to barriers, including high costs, restrictive healthcare policies, and limited awareness among clinicians. Many patients, particularly those from lower socioeconomic backgrounds, are unable to access GLP-1RAs despite their proven benefits. This disparity represents a significant gap in care that must be addressed to ensure equitable access to effective treatments.

Patients with SPMI should have the option to choose evidence-based treatments, including GLP-1RAs, to manage AIWG. Increasing access and implementing clear prescribing guidelines is important, as current limitations effectively restrict SPMI patients from obtaining a potentially effective treatment.

Healthcare providers, policymakers, and stakeholders must collaborate to expand equitable access to GLP-1RAs. In October 2024, the Quebec Liberal Party petitioned the Quebec National Assembly to include provincial coverage of weight-loss medications in general.^29^ This policy would ensure equitable access to the latest advances in metabolic and psychiatric care. By increasing accessibility, we can improve both physical and mental health outcomes for patients with SPMI, enhancing their quality-of-life and reducing the long-term costs associated with managing the complications of AIWG.^30^
